# Genome-Wide Analysis of the Lateral Organ Boundaries Domain Gene Family in Brassica Napus

**DOI:** 10.3390/genes11030280

**Published:** 2020-03-06

**Authors:** Tao Xie, Lei Zeng, Xin Chen, Hao Rong, Jingjing Wu, Jacqueline Batley, Jinjin Jiang, Youping Wang

**Affiliations:** 1 Jiangsu Provincial Key Laboratory of Crop Genetics and Physiology, Yangzhou University, Yangzhou 225009, China; d160113@yzu.edu.cn (T.X.); 18752782420@163.com (L.Z.); 18705275569@163.com (X.C.); d160114@yzu.edu.cn (H.R.); 18762714820@163.com (J.W.); wangyp@yzu.edu.cn (Y.W.); 2 School of Biological Sciences, University of Western Australia, Perth, WA 6009, Australia; jacqueline.batley@uwa.edu.au

**Keywords:** *Brassica napus*, *LATERAL ORGAN BOUNDARIES-domain* gene, comparative studies, conserved synteny, gene expression pattern

## Abstract

The plant specific LATERAL ORGAN BOUNDARIES (LOB)-domain (LBD) proteins belong to a family of transcription factors that play important roles in plant growth and development, as well as in responses to various stresses. However, a comprehensive study of LBDs in *Brassica napus* has not yet been reported. In the present study, 126 *BnLBD* genes were identified in *B. napus* genome using bioinformatics analyses. The 126 BnLBDs were phylogenetically classified into two groups and nine subgroups. Evolutionary analysis indicated that whole genome duplication (WGD) and segmental duplication played important roles in the expansion of the *BnLBD* gene family. On the basis of the RNA-seq analyses, we identified *BnLBD* genes with tissue or developmental specific expression patterns. Through *cis*-acting element analysis and hormone treatment, we identified 19 *BnLBD* genes with putative functions in plant response to abscisic acid (ABA) treatment. This study provides a comprehensive understanding on the origin and evolutionary history of *LBDs* in *B. napus*, and will be helpful in further functional characterisation of *BnLBDs*.

## 1. Introduction

The *LATERAL ORGAN BOUNDARIES (LOB)-domain (LBD)/ASYMMETRIC LEAVES2-LIKE (ASL)* gene is a newly discovered transcription factor gene family, playing important roles in various aspects of plant growth and development [1,2,3]. The first *LBD* gene, named *LOB*, was found based on the expression analysis of an enhancer trap line [1]. Hitherto, a total of 43 *LBD* genes have been identified in *Arabidopsis*, which were divided into two classes. The *LBD* genes in Class I contain a highly conserved CX_2_CX_6_CX_3_C (X are residues not conserved) zinc-finger-like motif (C block), a GAS (Gly-Ala-Ser) block, and a LX_6_LX_3_LX_6_L leucine zipper-like coiled-coil motif, whereas those in Class II only contain a C block [1]. The C block is approximately 22 amino acids in length, and is associated with DNA-binding. The GAS block is a conserved motif starting with a FX_2_VH motif and ending with a DP (V/I)G motif. The GAS motif and leucine zipper-like motif are required for protein–protein interaction of LBDs [1,3].

Following the identification of LBD genes in *Arabidopsis*, LBDs have been found in other plant species, such as *Oryza sativa*, *Malus domestica*, *Zea mays*, *Vitis vinifera*, *Morus notabilis*, *Lotus japonicas*, *Medicago truncatula*, *Brachypodium distachyon*, *Glycine max*, and *Eucalyptus grandis*. The number of LBD members ranged from 28 to 90 in different plants [4,5,6,7,8,9,10,11,12,13]. However, LBDs have never been identified in any species other than plants, indicating that LBD is a plant-specific gene family [1].

*LBDs* play important roles in the development of lateral organs, including leaf, root, flower, and embryo development [3]. *ASYMMETRIC LEAVES2* (*AS2*) in *Arabidopsis thaliana* is involved in the formation of symmetric flat leaf lamina. Overexpression of *AtAS2* resulted in upwardly curled leaves that fail to expand [14,15,16], and led to an abnormal adaxial–abaxial pattern via repression of the expression of polarity genes [17]. Overexpression of *LBD3* in rice also resulted in narrow and adaxially rolled leaves [18,19]. *INDETERMINATE GAMETOPHYTE1* (*IG1*) in maize was shown to be required for leaf and embryo sac development by affecting the *KNOTTED-LIKE HOMEOBOX* (*KNOX*) gene expression [18]. In addition, *ADVENTITIOUS ROOTLESS1* (*ARL1*), *LBD16*, *LBD29*, *LBD18*, and *LBD14* have been reported to regulate lateral root formation in *Arabidopsis* or rice [20,21,22,23,24]. Repression of *OsIG1* affects the number of female gametophyte and flower organs [25].

In addition to the functions in lateral organ development, *LBDs* are also involved in the regulation of anthocyanin and nitrogen metabolism, phytohormone accumulation (e.g., auxin, cytokinin, gibberellin (GA), and brassinosteroid), as well as response to environmental stimuli [3,26]. Mutation of *AtLBD25* reduced auxin sensitivity and lateral roots, and resulted in abnormal hypocotyl elongation under dark conditions [27]. Thatcher et al. first revealed that *AtLBD20* is regulated by jasmonate acid (JA) signaling, and *lbd20* mutant lines showed increased survival after *Fusarium oxysporum* infection [28]. In banana fruit, MaLBD5 was induced by cold temperature and methyl jasmonate (MeJA) treatment, and was involved in the transcriptional regulation of MeJA-mediated cold tolerance [29].

The conserved domain and gene structure of *LBDs* in Class II are different from those of Class I, thus the functions of *LBDs* in different classes are also different. In *Arabidopsis*, *LBD37*, *LBD38*, and *LBD39* repressed the anthocyanin synthesis by suppressing the key transcriptional factors (PAP1 and PAP2) of the flavonoid biosynthesis pathway, and affected the nitrogen metabolism by repressing many N-responsive genes [30,31]. Overexpression of *OsLBD37* and *OsLBD38* delayed the heading date and improved the crop yield in rice [32].

*B. napus* (AACC, 2n = 38) is a natural polyploid formed ~7500 years ago, which was derived from natural hybridization between *B. rapa* (AA, 2n = 20) and *B. oleracea* (CC, 2n = 18) [33]. Gene duplication plays an important role in plant evolution, which greatly contributed to the diversification of gene families, enlarged the genome size, and caused the genome complexity, as well as facilitated the formation of new species with better adaptation, such as species with improved disease resistance and tolerances to complex abiotic and biological stresses [34,35]. As reported, each Brassicaceae species has undergone gene duplication events during evolution [33,36,37]. Chalhoub et al. completed the whole genome sequencing and assembly of *B. napus*, and 101,040 gene models were generated from 35.5 Gb of seqencing data [33]. The assembled C subgenome (525.8 Mb) is bigger than A subgenome (314.2 Mb) [33]. The duplication history makes *B. napus* a model species for plant polyploidization research, and the gene expansion and contraction during rapeseed polyploidization would be interesting for gene diversification. How the LBDs are involved in the polyploidization of *B. napus* would also be of interest. The completion of the whole genome sequence of *B. napus* greatly facilitated the systematic analyses on the formation, evolution, and function of *LBDs* in *B. napus*. In this study, we comprehensively analyzed the structure, conserved motifs, chromosomal location, subcellular localization, and expression pattern of 126 LBDs in *B. napus*, as well as their synteny during genome duplication. This study provides an important basis for future functional studies of *LBDs* and may give new insights into improving the disease resistance and stress tolerance of *B. napus*.

## 2. Materials and Methods

### 2.1. Retrieval of LBDs in B. napus

The peptide sequence of *B. napus* was downloaded from the Genoscope database (http://www.genoscope.cns.fr/brassicanapus) [33]. The 43 *Arabidopsis* LBDs were acquired from the Plant Transcription Factor Database (http://planttfdb.cbi.pku.edu.cn/) [38]. We applied two methods to find all the LBDs in *B. napus*; firstly, the AtLBDs were used for protein-blast with an e-value of 1×e^−10^ against the peptide sequences of *B. napus*. Secondly, the conserved domain of LBDs (LOB domain, DUF260, pfam number: pfam03195) acquired from Pfam (http://pfam.xfam.org/) was used for blast to identify the BnLBDs with DUF260 as a query [39]. Then, the LOB domain in the predicted BnLBDs was screened again with Batch CD-Search (https://www.ncbi.nlm.nih.gov/cdd/) and HMMER (https://www.ebi.ac.uk/Tools/hmmer/) [2,40], and only the sequences with an LOB domain and complete C motif in the N-terminus were used for further analysis. The molecular weight and theoretical pI of the BnLBDs were calculated using the protein isoelectric point calculator (http://isoelectric.org/).

### 2.2. Phylogenetic Analysis and Characterization of LBD Proteins

The full length protein sequences of LBDs were aligned using the clustalX program (http://www.clustal.org/clustal2/) and edited by Jalview (http://www.jalview.org/). The sequence logos were drawn with WebLogo 3 (http://weblogo.threeplusone.com/). Phylogenetic trees were constructed with the neighbor-joining (NJ) method of MEGA 6.0 (https://www.megasoftware.net/) using the p-distance and pairwise deletion option. The tree reliability was assessed using 1000 bootstrap replications. In the present study, a phylogenetic tree of LBD proteins in *B. napus* and *A. thaliana* and a tree of BnLBDs, respectively, were generated.

The exon–intron structures of the *BnLBD* genes were extracted from the *B. napus* genome (http://www.genoscope.cns.fr/blat-server/cgi-bin/colza/webBlat). Multiple Em for Motif Elicitation (MEME, http://meme-suite.org/tools/meme) was used to analyze the conserved motifs in BnLBD proteins. The gene structure and the conserved motifs were visualized using the Amazing Optional Gene Viewer in TBtools [41].

### 2.3. Cis-Acting Element Analysis

To analyze the *cis*-acting elements of *BnLBDs*, TBtools was used to obtain the 2000 bp of genomic DNA sequence upstream of coding sequences (CDS). The PlantCARE software (http://bioinformatics.psb.ugent.be/webtools/plantcare/html/) (Gent, Belgium) was used to analyze the presence of different *cis*-acting elements.

### 2.4. Chromosomal Location, Identification of Orthologous and Paralogous LBD Genes

The length of each chromosome and the location of each *BnLBD* gene were retrieved from the Genoscope database. *BnLBDs* were localized on the chromosomes using Map Gene 2 Chromosome v2 (MG2C) (http://mg2c.iask.in/mg2c_v2.0/). Multiple collinear scanning toolkits (MCScanX) were used to analyze gene replication events and synteny relationships among *B. napus*, *A. thaliana*, *B. rapa*, and *B. oleracea*. To acknowledge the relationship of paralogous *LBDs* in *B. napus*, and the orthologous of *LBDs* among *B. napus* and other species, the syntenic maps were constructed with the Amazing Super Circos software and the Dual Systeny Plotter in TBtools, respectively [41]. The synonymous rate (Ks), non-synonymous rate (Ka), and Ka/Ks ratio of each gene pair were calculated using KaKs Calculator 2.0 [42].

### 2.5. Subcellular Localization Analysis of BnLBD Proteins

The subcellular localization of all the BnLBD proteins was predicted with Plant-mPLoc (http://www.csbio.sjtu.edu.cn/bioinf/plant-multi/) (San Diego, CA, USA) and ProtComp v.9.0 in softberry (http://linux1.softberry.com/) (Stockholm, Sweden). In order to verify the predicted results, the coding sequence of BnLBD46, BnLBD104, and BnLBD105 was amplified from the cDNA of *B. napus* cv. Damor-*bzh*, and cloned into a green fluorescent protein (GFP) fusion expression vector pMDC83 via an enzyme (*Spe*I/*Asc*I) digestion-ligation method. All the primers were synthesized by TSINKE Biotech and are listed in Table S1. The *Agrobacterium tumefaciens* strain GV3101 harboring the *35S:BnLBD-GFP* constructs were injected into the abaxial epidermis of *Nicotiana benthamiana* leaves for transient expression [43]. The florescence images were captured using a confocal laser-scanning microscope (TCS SP8 STED, Leica, Germany).

### 2.6. Plant Material and Stress Treatment

To investigate the expression pattern of *BnLBDs*, three replicates of fifteen samples representing the major developmental tissues and organs of *B. napus* line ‘J9712′ were collected for RNA-seq analysis, including leaf near bolting stage; cotyledon; hypocotyl; root; stem; shoot apical meristem (SAM); 3 mm bud; 6 mm bud; endosperml silique at 14 days after pollination (DAP); and five seed samples at 21, 28, 35, 42, and 50 DAP. Using the RNA-seq data, a heat map of *BnLBDs* in different developmental stages was generated based on the log10 transformed values of FPKM (fragments per kilobase of transcript per million fragments mapped) values, and if FPKM = 0, then log_10_FPKM = −3. To investigate the response of *BnLBD* gene family under abscisic acid (ABA) treatment, the five-week-old seedlings of *B. napus* line ‘J9712′ were treated with 100 μM ABA. Samples were collected at 0 h, 1 h, 3 h, 6 h, and 12 h after treatment. Six leaves were pooled from three seedlings, and stored at −80 °C for further gene expression analysis.

### 2.7. RNA Extraction, cDNA Synthesis, and Quantitative Real Time PCR Analysis

Total RNA of leaf samples under ABA treatment was extracted according to the protocal of RNA isolater Total RNA Extraction Reagent (Vazyme, China). The total RNA was reverse transcribed into cDNA using HiScript III RT SuperMix for qPCR (Vazyme, China). qRT-PCR was performed on StepOnePlus Real-Time PCR Syetem (Thermo, Waltham, MA, USA) using PowerUp SYBR Green Master Mixes (Thermo, Waltham, MA, USA). The 2^-△△Ct^ method was used to calculate the relative gene expression levels of *BnLBD* genes. All the primers were synthesized by TSINKE Biotech and are listed in Table S1.

## 3. Results

### 3.1. Identification of BnLBD Proteins

A total of 139 BnLBD proteins were identified in *B. napus*, including 135 BnLBDs identified using AtLBDs as queries and 136 BnLBDs screened with the LOB domain as query. However, six BnLBDs were excluded because of the absence of an LOB domain and seven BnLBDs were removed for lack of a complete C block in the N-terminus. Thus, we finally identified 126 BnLBD proteins, and these proteins were named with serial numbers from BnLBD1 to BnLBD126 depending on their location on the chromosomes. As *B. napus* was derived from natural hybridization between two diploids (*B. rapa* and *B. oleracea*), while the diploids in *Brassica* underwent polyploidization events based on *A. thaliana*, the number of LBDs in *B. napus* is much less than the theoretical value after the triplication and duplication events, and ~51.16% LBDs might be lost during *B. napus* evolution. The detailed information of each BnLBD protein is listed in Table S2, including gene identifier (gene ID), genomic location, protein length, isoelectric point (pI), and molecular weight (Mw). The BnLBD genes consisted of 104–358 amino acids (AA) with an average length of 214 AA, while the molecular weight ranged from 11.15 to 40.60 kDa and the pI of BnLBD proteins ranged from 4.73 to 9.08.

**Figure 1:**
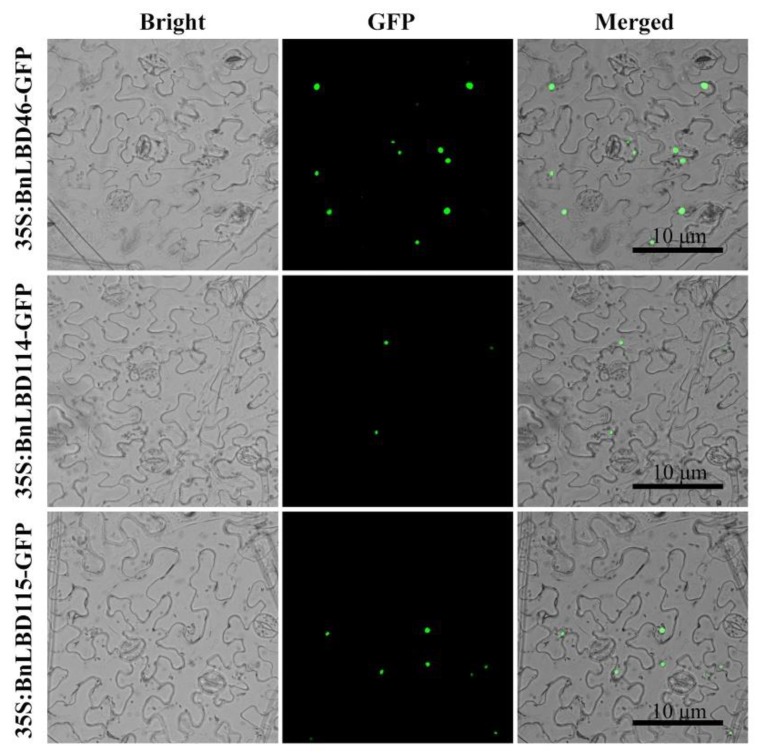
Subcellular localization of BnLBD46/114/115-GFP. GFP fluorescence is shown in green. Bars = 10 μm. LBD, LATERAL ORGAN BOUNDARIES (LOB)-domain; GFP, green fluorescent protein.

Using Plant-mPLoc and ProtComp to predict the subcellular location of 126 BnLBD genes, we found most of them were located in the nucleus. To validate the predicted results, transient expression of BnLBD46, BnLBD104, and BnLBD105 fused with GFP was performed in tobacco, and the subcellular localization of these proteins was consistent with the predicted results (Figure 1).

### 3.2. Sequence Alignment and Phylogenetic Analysis of LBD Proteins

**Figure 2:**
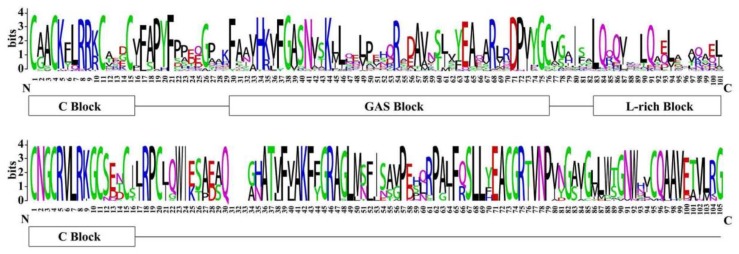
Sequence logos of LOB domains in BnLBD proteins. Multiple alignment analysis of LOB domains was performed with Clustal X. The sequence logos were drawn by WebLogo 3. The bit score exhibits the information content for each position in the sequence. GAS, Gly-Ala-Ser.

A protein sequence alignment was performed to identify the conserved amino acids and classify the BnLBD proteins. As shown in Figure 2 and Figure S1, a completely conserved CX_2_CX_6_CX_3_C zinc-finger-like motif (C block) existed in all BnLBDs, as reported in other plant species. A further two conserved motifs, a LX_6_LX_3_LX_6_L leucine zipper-like coiled-coil motif and a GAS (Gly-Ala-Ser) block between the C block and the leucine zipper-like motif, were identified in most BnLBDs. Generally, 104 BnLBD proteins with C block, GAS block, and the leucine zipper-like motif were classified into Class I, while 22 BnLBDs with only a C block were classified into Class II of BnLBDs.

**Figure 3:**
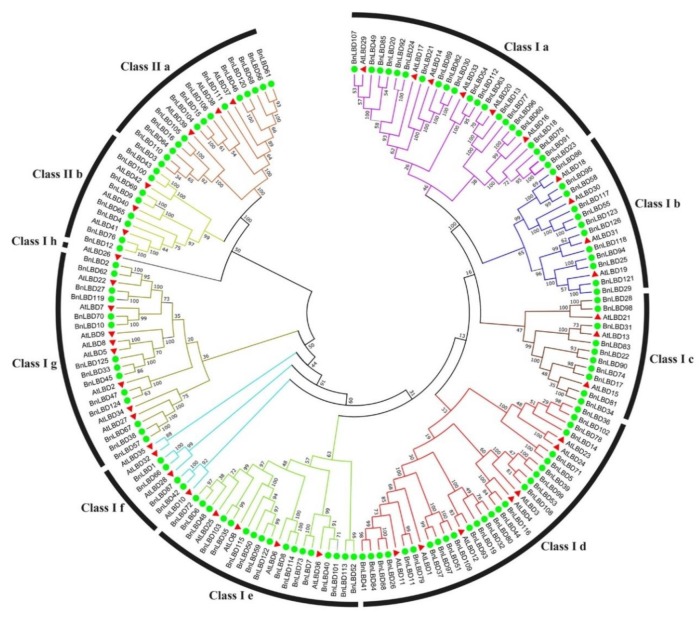
Phylogenetic relationship of LBD proteins in *B. napus* and *A. thaliana*. The different colors indicate different subgroups of the LBD family. The green circles and red triangle represent LBDs from *B. napus* and *A. thaliana*, respectively. The numbers on the branches indicate the reliability percent of bootstraps value based on 1000 replications.

To understand the evolutionary relationship of BnLBDs, a phylogenetic tree was constructed based on the alignment of the BnLBD and AtLBD protein sequences. As shown in Figure 3, the LBD proteins were classified into two groups (Class I and Class II). The larger group (Class I) was further divided into eight subgroups (Class Ia–Class Ih) and Class II was divided into two subgroups (Class IIa and Class IIb). Among the 126 BnLBDs and 43 AtLBDs, 104 BnLBDs and 37 AtLBDs with a complete C block, GAS block, and a leucine zipper-like motif were clustered in Class I, while 22 BnLBDs and 6 AtLBDs with a C block were clustered in Class II. Thirteen BnLBDs clustered in Class IIa were orthologs of AtLBD37, AtLBD38, and AtLBD39, which have been reported as negative regulators of anthocyanin biosynthesis and affect additional nitrogen responses in *Arabidopsis* [30].

### 3.3. Structural and Conserved Motif Analysis of BnLBD Genes

**Figure 4:**
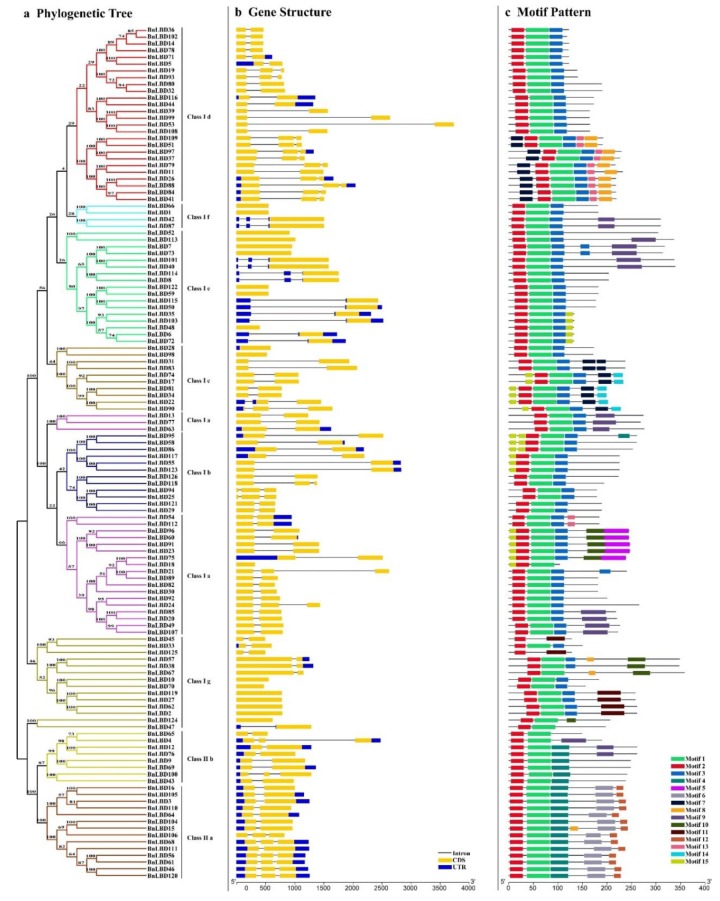
Phylogenetic relationships, gene structure, and motif composition analysis of the LBD family in *B. napus*. (**a**). The phylogenetic tree based on the full-length sequences of BnLBD proteins using MEGA 5 software. The different colors indicate different subgroups of BnLBDs. The number on the branches indicates the reliability percent of bootstraps value based on 1000 replications. (**b**). Exon/intron structure of BnLBDs. Blue boxes indicate 5′- and 3′-untranslated regions, yellow boxes indicate exons, and black lines indicate introns. The scale bar represents 0.5 kb. (**c**). The architecture of conserved protein motifs of LBD proteins. Motifs 1–15 are displayed in different colored boxes, and the motif structure is provided in Additional File 3. The scale bar represents 50 amino acids.

The alterations in exon–intron structure play an important role in gene function divergence, especially for the duplicate genes [44]. In this study, the exon–intron structure of 126 *BnLBD* genes was investigated (Figure 4b). Statistical analyses revealed that the exon number of *BnLBDs* ranged from one to four, of which 69 *BnLBDs* were identified with two exons, only one gene (*BnLBD100*) was found with four exons, and 24 *BnLBDs* contain three exons. The remaining 32 *BnLBDs* contain one exon, without any intron disrupting the coding sequence. In addition, the genes from the same phylogenetic groups from the same subfamily were more similar in exon–intron structure. For example, most genes in Class If and Class Ie had no intron in the coding sequence region, while a clade in Class Id and one clade in Class IIa contained three exons (Figure 4a, Figure 4b). We also found the length of exons is similar in genes from the same subfamily, but the length of introns varied a lot in some subfamilies, such as BnLBD39/99, BnLBD53/108, and BnLBD95/58, which may contribute to the functional divergence of the duplicated genes.

We used MEME to analyze the conserved motif of the 126 BnLBD proteins, and a total of 15 conserved motifs in BnLBDs were identified with a length ranging from 14 to 50 amino acids (Figure 4c, Figure S2). We found the structure of BnLBDs was similar in the same subgroup. The conserved motif 1 and motif 2 existed in most BnLBDs, except BnLDB45 and BnLDB125, which lack motif 1. Motif 3 was identified in BnLBDs clustered in Class I, motif 4 existed in Class II, while other motifs were identified in specific subgroups. For example, motif 5 and motif 12 were identified in Class Ia and Class IIa, respectively. Motif 7 was only identified in Class Ic and Class Id.

### 3.4. Chromosomal Distribution and Genomic Duplication of BnLBD Genes

**Figure 5:**
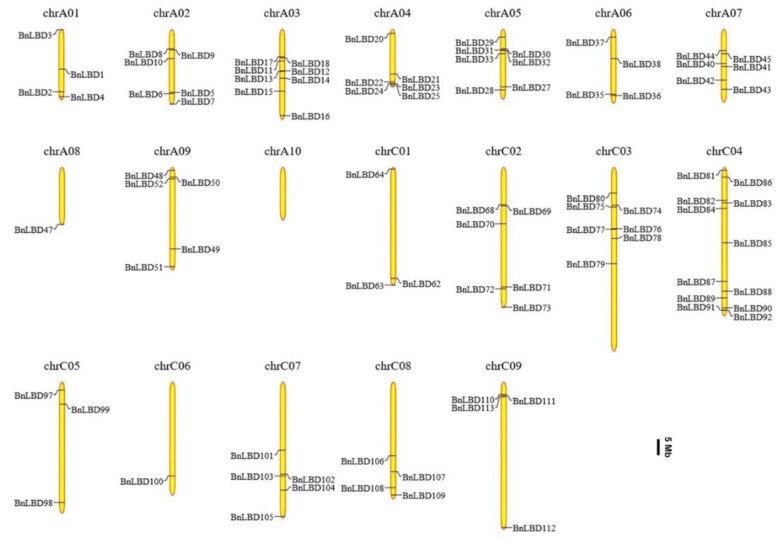
The distribution of *BnLBD* genes on the *B. napus* chromosomes. A total of 92 *BnLBD* genes are mapped to the 19 chromosomes, and the unmapped 34 *BnLBDs* are unassembled scaffolds. The chromosomal position of each *BnLBD* gene was mapped to the *B. napus* genome. The chromosome number is indicated at the top of each chromosome. The scale is in megabases (Mb).

As reported, both the A and C subgenomes of *B. napus* have undergone duplications [33,45]. Here, we mapped the *BnLBDs* onto the *B. napus* chromosomes, and found 92 *BnLBD* genes were unevenly distributed across the 19 chromosomes (Figure 5). The remaining 34 *BnLBD* genes (14 on Ann random chromosomes and 20 on Cnn random chromosomes) were not assigned to specific chromosomes, owing to the incomplete *B. napus* genome. A total of 61 and 65 *BnLBD* genes were located on A and C subgenomes, respectively. Chromosome C04 contained the largest number of *BnLBD* genes (12 genes), followed by Chromosome A03, A05, and C03, with 8, 7, and 7 *BnLBDs*, respectively. Only one *BnLBD* was assigned on Chromosome A08 and C06, and no *BnLBD* was identified on Chromosome A10. The number of *BnLBDs* was not positively correlated with the chromosome length. According to previous studies, the *LBD* genes tended to be located on both ends of chromosomes. However, this was not observed in the chromosomal distribution of *BnLBDs*.

**Figure 6:**
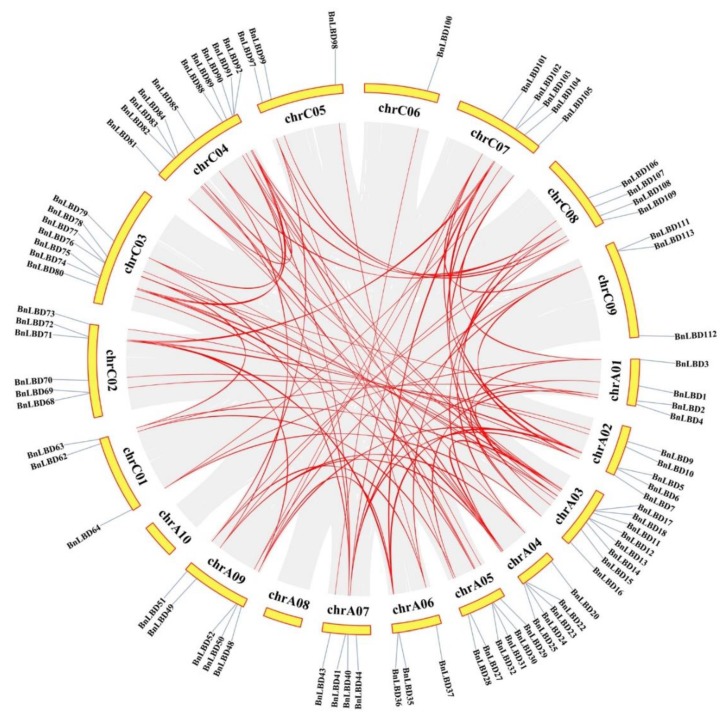
Chromosomal distribution and interchromosomal relationships of *BnLBD* genes. Gray lines indicate all synteny blocks in the *B. napus* genome, and the red lines indicate duplicated *BnLBD* gene pairs. The chromosome number is indicated on the top of each chromosome.

**Figure 7:**
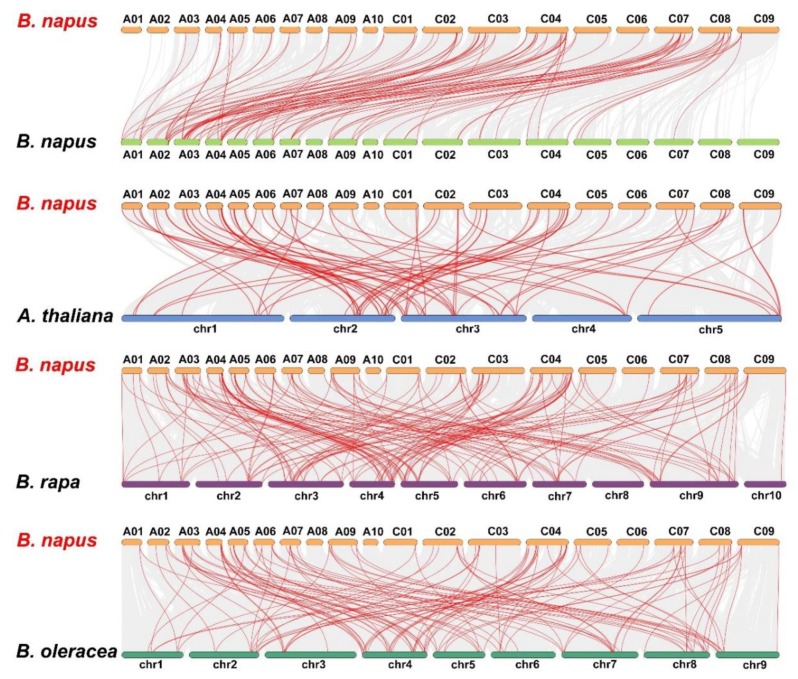
Synteny analysis of *LBD* genes in *B. napus* and three ancestral plant species. Gray lines in the background indicate the collinear blocks within *B. napus* and other plant genomes, while the red lines highlight the syntenic *LBD* gene pairs.

Gene duplication is universal in all organisms and important in dissecting the novelties in plant evolution [46]. Thus, we investigated the duplication events of the *LBD* gene family in *B. napus* (Figure 6, Figure 7), and found that all the 126 *BnLBDs* were results of duplication events (Table S3), of which 93 *BnLBDs* were derived from whole-genome duplication (WGD) or segmental duplications, and the other 33 *BnLBD* genes were results of dispersed duplications. Using MCScanX software, 104 paralogous gene pairs were identified (Table S4). These results indicated that gene duplication largely contributed to the expansion of *LBD* genes in *B. napus* genome, and the WGD or segmental duplication events played the main driving role.

To understand the evolution of the *BnLBD* gene family in the Brassicaceae, we analyzed the syntenic relationships between *B. napus* and *Arabidopsis*, *B. napus* and *B. rapa*, and *B. napus* and *B. oleracea*. Collinearity analysis revealed that a large number of orthologous *LBDs* existed in *B. napus* compared with *B. rapa*, *B. oleracea*, and *Arabidopsis* (Figure 7, Table S4). Generally, 100 of the 126 *BnLBDs* (79.37%) had a syntenic relationship with *LBDs* in other species, of which 82 *BnLBD* genes showed a syntenic relationship with *Arabidopsis*, 95 *BnLBDs* were syntenic to *B. rapa*, and 78 *BnLBDs* had synteny to *B. oleracea*. We found 86 *BnLBD* genes were directly inherited from *BoLBDs* (42 genes) and *BrLBDs* (44 genes). The synteny analysis indicates that the expansion of the *LBD* gene family in *B. napus* was mainly the result of whole-genome duplication.

To characterise the selective pressure on duplicated *BnLBD* genes during the evolutionary process, Ks, Ka, and Ka/Ks ratios were calculated for the paralogous gene pairs in *B. napus*, and the orthologous gene pairs among *B. napus*, *B. rapa*, *B. oleracea*, and *A. thaliana* [47]. We found most of the *BnLBD* gene pairs with Ka/Ks < 0.5, indicating that the *BnLBD* gene family might have undergone intense purifying selection in the evolutionary process (Table S3, Figure S3). The divergence time of homologous gene pairs between *B. napus*–*B. napus*, *B. napus*–*B. rapa*, *B.napus*–*B. oleracea*, and *B. napus*–*A. thaliana* peaked at 0.11, 0.02, 0.01, and 0.14 (Figure S3), respectively, which means that the divergence of *LBD* gene pairs in *B. napus* and *A. thaliana* was earlier.

### 3.5. Tissue Expression Analysis of BnLBD Genes

**Figure 8:**
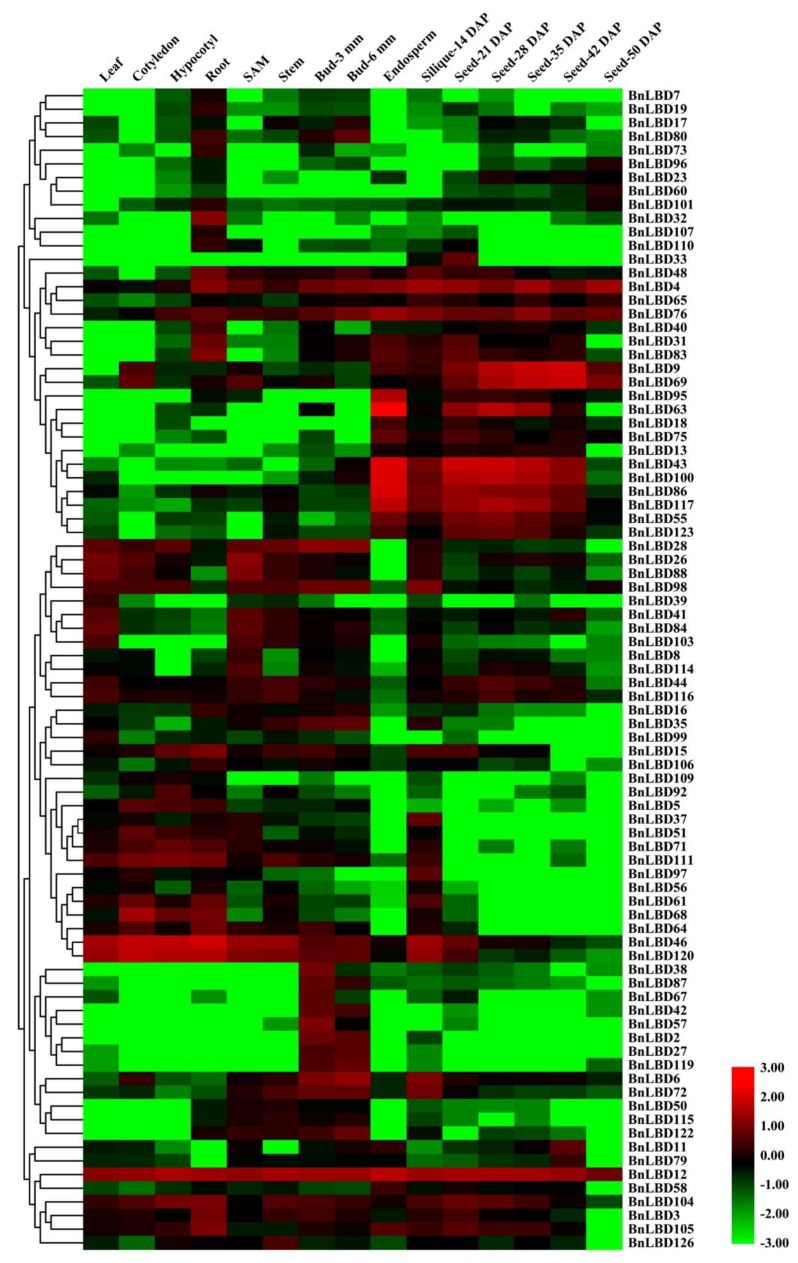
Expression pattern of *BnLBD* genes in different tissues and developmental stages. The heatmap is generated based on log10 transformed values of FPKM (the reads per kilobase per million mapped reads) values, and if FPKM = 0, then log_10_FPKM = −3. *BnLBD* genes are clustered according to hierarchical clustering. FPKM, fragments per kilobase of transcript per million fragments mapped; SAM, shoot apical meristem; DAP, days after pollination.

On the basis of the high-throughput RNA sequencing data of different tissues and organs at different development stages of *B. napus*, we analyzed the tissue specific expression pattern of *BnLBD* genes, and found 41 *BnLBD* genes were not or were weakly expressed in all tissues (Figure 8, Table S5). We found all *BnLBD* genes in Class II and 63 of the 104 *BnLBD* genes in Class I were expressed in *B. napus*. Generally, the expression pattern of *BnLBD* genes could be roughly divided into four types. The first type had low expression in all tissues. The second type was expressed in almost all tissues, such as *BnLBD4*, *BnLBD12*, *BnLBD76*, *BnLBD46*, and *BnLBD120*. The third type was expressed in most tissues, but was highly expressed in specific tissues, such as *BnLBD28*, *BnLBD26*, *BnLBD88*, and *BnLBD98*. The fourth type was only expressed in one or two tissues. For instance, *BnLBD38*, *BnLBD87*, *BnLBD67*, *BnLBD42*, *BnLBD57*, *BnLBD2*, *BnLBD27*, and *BnLBD119* were only expressed in bud, while *BnLBD9*, *BnLBD69*, *BnLBD63*, *BnLBD43*, *BnLBD100*, *BnLBD86*, *BnLBD117*, *BnLBD55*, and *BnLBD123* were only expressed in the endosperm or seeds at developmental stages. In addition, the expression level of the *LBD* gene in Class II was generally higher than that in Class I, indicating that the *LBD* gene in Class II might be more important for the growth and development of rapeseed.

### 3.6. Cis-Acting Element Analysis of BnLBDs

**Figure 9:**
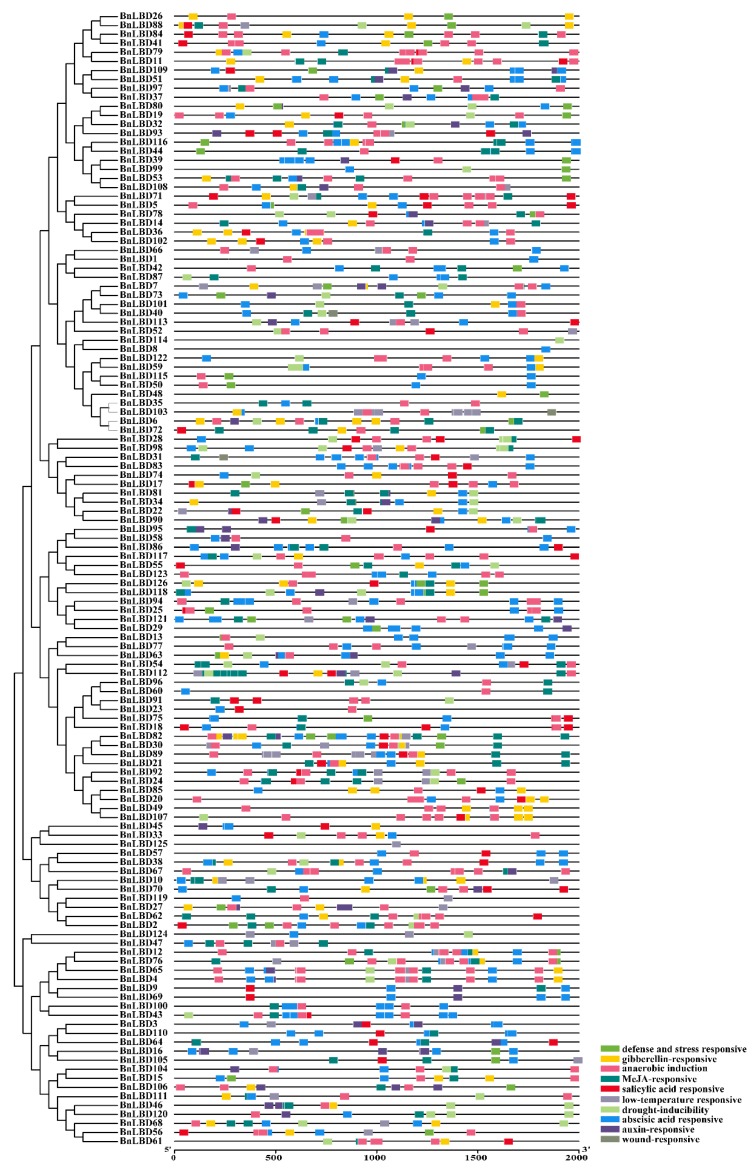
*Cis*-element analysis on the promoter regions of *BnLBD* genes. The presence of different *cis*-acting elements was determined by the PlantCARE software. Different colored boxes represent different *cis*-elements.

*Cis*-acting elements play an important role in regulating gene expression, and genes with similar functions may contain the same regulatory elements in their promoters. The *cis*-elements in 126 *BnLBD* genes were analyzed using PlantCARE software, and four types of *cis*-acting elements were found in the promoter region of the *BnLBD* gene family, including elements related to plant growth and development, abiotic stress responses, hormones responses, and basic promoter elements in eukaryotes (such as CAAT-box and TATA-box) (Figure 9, Table S6). Promoter elements associated with growth and development mainly include GCN4-motif and AACA-motif involved in endosperm expression; CAT-motif related to meristems expression; and a large number of light-response elements such as Box 4, GA-motif, G-box, and TCT-motif. We found the elements related to light response were most common motif in *BnLBD* promoters, indicating that the *BnLBD* gene family could be induced by light to regulate plant growth and development. In the second category, mainly *cis*-elements related to GA, auxin, abscisic acid (ABA), MeJA, and salicylic acid (SA) responses were identified, of which we found the motifs associated with the ABA response were the most abundant, followed by motifs associated with the MeJA response. In addition, we also found motifs related to stresses responses, such as low temperature response (LTR) involved in low temperature response, MYB binding site (MBS) for drought induction, wound-responsive element (WUN-motif) associated with wound response, and anaerobic regulatory element (ARE) essential for anaerobic induction. Overall, these results indicate that *BnLBDs* might play functions in regulating plant growth and development, response to abiotic stresses, and hormone responses. In addition, we found most *BnLBD* genes with different types of *cis*-element in the promoter regions, suggesting that these *BnLBDs* may be involved in various biological processes and regulatory pathways.

### 3.7. The Expression Pattern of BnLBD Genes under ABA Treatment

**Figure 10:**
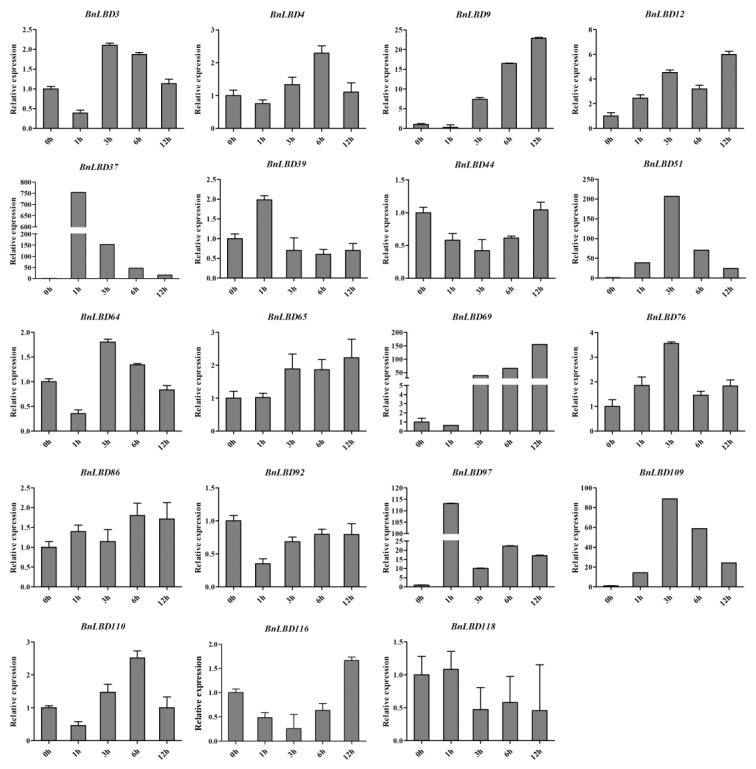
The expression pattern of *BnLBD* genes under abscisic acid (ABA) treatment. Data were normalized to *BnActin 2* gene and vertical bars indicate standard deviation.

Phytohormones are essential for plant growth and development, and play important roles in stress response [48]. Several *LBD* genes have been identified with functions in response to hormone treatments and abiotic stresses. As we found the motifs related to ABA response were the most abundant *cis*-element in *BnLBD* promoters, we analyzed the expression pattern of *BnLBDs* under ABA treatment. A total of thirty *BnLBDs* with more than five ABA-responsive elements in the promoter regions were analyzed, of which *BnLBD37/51/97/109* (orthologous with *LBD1* in *A. thaliana*) and *BnLBD9/69* (orthologous with *LBD40* in *A. thaliana*) were significantly up-regulated after ABA treatment. The expressions of *BnLBD9* and *BnLBD69* were up-regulated under 3 h of ABA treatment, and the expression level under 12 h of ABA treatment was ~25 and 150 times higher than that of the control group, respectively. The expression of *BnLBD51* and *BnLBD109* was significantly up-regulated under 3 h of ABA treatment. *BnLBD37* and *BnLBD97* were strongly induced by 1 h of ABA treatment (Figure 10). Besides, we found another 11 *BnLBD* genes that were not induced by ABA treatment. These results indicate that 19 members of the *BnLBD* gene family might be involved in the ABA signaling pathway, which is consistent with the result of *cis*-element analysis in the promoters.

## 4. Discussion

The *LBD* genes encode a class of plant-specific transcription factors, which play important roles in regulating the plant growth and development, especially in the development of lateral organs [1,14,15]. The *LBD* gene family has been extensively studied in different plants species, but the comprehensive analysis of *LBDs* in the oil crop *B. napus* has not yet been reported. In this study, we identified 126 *BnLBD* genes in *B. napus*. These *BnLBD* genes were divided into two groups and ten subgroups based on the evolutionary relationships, which is highly consistent with the reports in *Arabidopsis*, *G. max*, and *Z. mays* [1,6,12]. The variations in the structure of *BnLBDs* might be because of the gain or loss of introns/exons in the long-term evolutionary process. On the basis of the conserved motif analysis of BnLBDs, we suspect that group-specific and subgroup-specific motifs might be responsible for the functional divergence of the *BnLBD* gene family. In addition, the similar motif structure, exon–intron pattern, and protein sequences in each subgroup of BnLBDs might indicate the gene replication events during the evolutionary process.

*B. napus*, as an allopolyploid, was formed by natural hybridization between *B. rapa* and *B. oleracea* [33]. There are two main mechanisms for gene duplication, WGD /segmental duplication and tandem duplication [49]. WGD/segmental duplication is very common in plant genomes, as most plants have undergone polyploidy events during evolution, and thus retain many duplicated chromosome segments in their genomes [50,51]. Compared with other plant species such as *Arabidopsis*, *O. sativa*, *Z. mays*, *G. max*, and *Populus*, which contain 43, 35, 44, 80, and 57 *LBD* genes [1,6,12,13], respectively, we found *B. napus* with the largest number of *LBD* genes, indicating the genome duplication during evolution of *B. napus* greatly contributed to the diversification of *BnLBDs*. Further, all 126 *BnLBD* genes were identified as the result of gene duplication, 93 of them were identified with WGD or segmental duplications history, and the rest resulted from dispersed duplications. This indicated that WGD and segmental duplications are the primary force for the expansion of the *BnLBD* gene family.

In addition, tandem replication is another type of gene replication characterized by the presence of multiple members of a gene family in the same or adjacent intergenic regions [49]. In this study, we conducted a genome-wide analysis to identify all tandem replication gene pairs, and 3149 gene pairs were identified with tandem duplication, accounting for ~6% of the genes in the *B. napus* genome. Unfortunately, none of the LBD family members were found in these gene pairs. Additionally, 33 *BnLBD* genes were identified with dispersed duplication. Hitherto, little research on dispersed gene duplication has been reported in plant species. In the human genome, dispersed duplications such as transposon insertion and copy number variation are very common [52]. In heteroploid plants, the expansion of the genome is usually accompanied by the insertion of transposon factors [53,54]. Transposable elements in *Brassica* species are also abundant [54,55]. However, whether these transposons are one of the reasons for the expansion of *LBD* gene family is still unclear.

After gene duplication, not all the duplicated genes are retained or keep the original function. In theory, non-functionalization, neofunctionalization, and subfunctionalization are the three possible evolutionary consequences for duplicated genes [46]. Comparative genomic studies have shown that *Brassica* species, such as *B. rapa* and *B. oleracea*, diverged from a common ancestor *A. thaliana* via whole-genome triplication (WGT) approximately 20 to 40 million years ago [33,36,56]. Thus, three copies of each *A. thaliana* gene should exist in *B. rapa* and *B. oleracea*. The *Arabidopsis* genome contains 43 *LBD* genes, therefore, *B. rapa* and *B. oleracea* should contain more than 120 *LBD* genes after WGT, and ultimately result in even more *BnLBD* genes in *B. napus*. However, only 126 *BnLBD* genes were identified in this study, indicating that more than half of the *LBD* genes were lost after WGT. As reported, *B. napus* was derived from natural hybridization between two diploids (*B. rapa* and *B. oleracea*), while the diploids in *Brassica* have undergone polyploidization events based on *Arabidopsis thaliana*. Thus, we compared *B. napus* with *A. thaliana* to analyze the gene loss after WGD, and found 38.02% of genes were lost after WGD in *B. napus* (27,169 in *A. thaliana* and 101,040 in *B. napus*), while 51.16% of LBDs were lost in *B. napus* (43 in *A. thaliana* and 126 in *B. napus*). Compared with the gene loss rate in the whole genome, we may speculate the BnLBDs have undergone strong selection during *B. napus* evolution, and the LBDs kept should have interesting functions in *B. napus* development. On the basis of the phylogenetic tree, we can see that most *AtLBD* genes have more than one orthologous gene in *B. napus*, indicating that the *LBD* gene family has been expanded. However, most *AtLBD* genes have less than six orthologous genes in *B. napus*, which was less than expected. This agreed with the shrink of gene number in *B. napus*, indicating the *BnLBD* gene family was contracted during *B. napus* diversification. Besides, the orthologs of six *AtLBDs* (*AtLBD8*, *AtLBD9*, *AtLBD26*, *AtLBD32*, and *AtLBD34*) were not found in the *B. napus* genome, indicating that all the duplicated copies of these *LBDs* were lost during the evolutionary process. These lost *LBDs* might be redundant genes that were gradually replaced by other genes with similar functions. Previous studies also showed that all *BnLBD* genes have undergone intense purification selection, which plays a key role in maintaining the number of genes (Table S2, Figure S3).

According to the tissue expression pattern of *BnLBDs*, we found that not all the remaining *BnLBD* genes could be expressed normally, and more than 40 *BnLBD* genes were not expressed in the 15 tissues we analyzed. We suspect these genes might be expressed at specific developmental stages that were not covered in this study, or they were silenced after WGT. Another possibility is that these genes are pseudogenes. In addition, neofunctionalization and subfunctionalization may also greatly contribute as evolutionary forces in the divergence of the *BnLBD* gene family. Most of the paralogs in the *BnLBD* gene family are highly conserved at both the DNA and protein levels, including gene structure, protein sequence, and motif composition. The differences existing in some *BnLBD* gene pairs would lead to gene functional divergence and help to drive new functions. In *Arabidopsis*, LBD37/38/39 have been confirmed with functions in regulating anthocyanin biosythesis, and they are transcription factors located in the nucleus. Subcellular localization of BnLBD46/104/105, which are homologous to LBD37/38/39, indicated they are also nucleus anchered proteins, indicating they may with similar functions as LBD37/38/39.

Some of gene pairs were identified with variations in gene structure, such as loss or gain of motif in BnLBD38/57 and BnLBD15/104, as well as segmental loss or gain of intron in BnLBD11/79 and BnLBD118/126. Furthermore, the differential expression pattern of duplicated *BnLBD* gene pairs also indicated that these genes might have undergone functional differentiations. For example, *BnLBD37* and *BnLBD51*, the orthologs of *AtLBD1*, were identified with different expression patterns in different tissues and under stress conditions. This indicates that *BnLBD37* and *BnLBD51* might have undergone functional divergence, and either generated new functions or inherited partial functions of ancestral genes. Besides, we found several couples of *BnLBD* homologs with different expression patterns during rapeseed development. For instance, we also found *BnLBD104* and *BnLBD106* (orthologous with *LBD38* in *A. thaliana*) with totally different expression patterns throughout rapeseed development, and *BnLBD12* and *BnLBD65* (orthologous with *AtLBD41* in *A. thaliana*) with different expression patterns in different developmental stages of *B. napus*.

Many studies have revealed that the *LBD* gene family plays significant roles in plant growth and development, as well as in responding to biotic and abiotic stresses [57,58]. On the basis of the tissue expression pattern, we found some valuable *BnLBD* genes might have functions in specific physiological processes. For instance, *BnLBD46/120* and *BnLBD15/104* were highly expressed in the root tissues, and their orthologous genes in *A. thaliana* (*AtLBD37* and *AtLBD38*) were also reported with high expression in root tissues and functioned as the repressors of N-repressed gene and important molecular components in plant NO3^−^/N signaling. This indicates that *BnLBD46/120* and *BnLBD15/104* may have the same function in *B. napus* [30,59].

The functional roles of *BnLBD* genes related to different stresses were also speculated in this study. On the basis of PlantCARE software, we identified a large amount of *cis*-acting elements, which were associated with abiotic stress responses and hormones responses. All *BnLBD* promoters contain at least one *cis*-element associated with abiotic stress or hormonal response, suggesting that these genes may play functions in plant response to abiotic stress and hormonal stress. To further confirm this speculation, we analyzed the expression pattern of *BnLBD* genes to ABA treatment, and found the expression level of 19 *BnLBDs* was significantly induced after ABA treatment. This indicates that these *BnLBD* genes play functions in plant response to ABA treatment and may participate in plant responses to biotic and abiotic stresses. In general, this study provided general information on the diversification and putative functions of *LBD* genes in *B. napus*, and would be helpful in further functional studies of *BnLBDs*.

## 5. Conclusions

In this study, we systematically analyzed 126 *LBD* genes in *B. napus*, which were further classified into nine subgroups, and each group was clustered with similar gene structure and motif composition. Collinear analysis confirmed the high homology among *BnLBDs* and *LBDs* in *A. thaliana*, *B. rapa*, and *B. oleracea*. The evolutionary analysis indicated that whole-genome duplication and segmental duplication are the primary sources for the expansion of *LBD* genes in the *B. napus* genome. After polyploidization, gene loss and functional divergence occurred in the *BnLBD* gene family. On the basis of tissue expression, we identified the *BnLBD* genes with putative roles in specific plant growth and developmental stages. Through promoter analysis and hormone treatment, we identified 19 *BnLBD* genes involved in plant response to ABA treatment. Generally, the bioinformatics and expression analysis of *BnLBDs* provided us with a basic, but comprehensive understanding of the origin and evolutionary history of *LBDs* in *B. napus*, and also provided a theoretical basis for the further functional study on *BnLBDs*.

## Supplementary Materials

The following are available online at https://www.mdpi.com/2073-4425/11/3/280/s1, Figure S1. Multiple sequence alignment of LOB domains in BnLBD proteins using ClustalX, Figure S2. Conserved protein motifs BnLBDs family. The bit score exhibits the information content for each position in the sequence, Figure S3. Ka/Ks value and divergence time distribution of *LBD* genes. (a). *B. napus*-*B. napus*. (b) *B. napus*-*B. rapa* (c). *B. napus*-*B. oleracea*. (d). *B. napus*-*A. thaliana*, Table S1. The primers used in this study, Table S2. The information of *BnLBD* gene family in *B. napus*, Table S3. Duplication type of *BnLBD* genes, Table S4. Ka/Ks calculation of the duplicated *LBD* gene pairs, Table S5. The RNA-seq data of *BnLBD* genes in different tissues and developmental stages, Table S6. The *cis*-elements on the promoter regions of *BnLBD* genes.
