# Supplementary figures and images for: Genome-Wide Analysis of the Lateral Organ Boundaries Domain Gene Family in Brassica Napus
Source: Genes (Basel). 2020 Mar 6;11(3):280. doi: 10.3390/genes11030280 (PMC7140802; doi:10.3390/genes11030280)

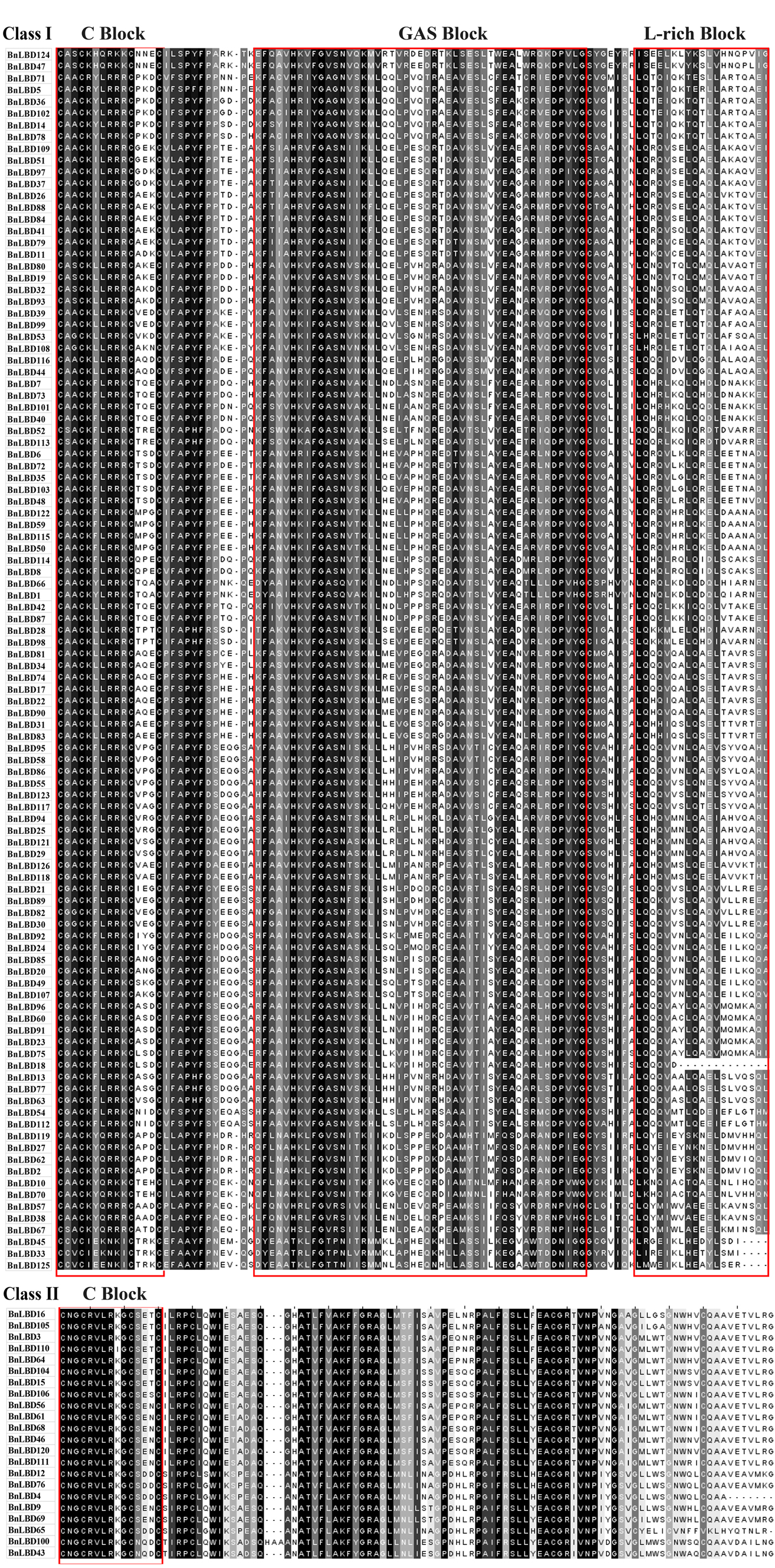

Supplement: Supplementary file 1 [file genes-11-00280-s001.zip › Additional files-20200306/Figure S1.jpg]

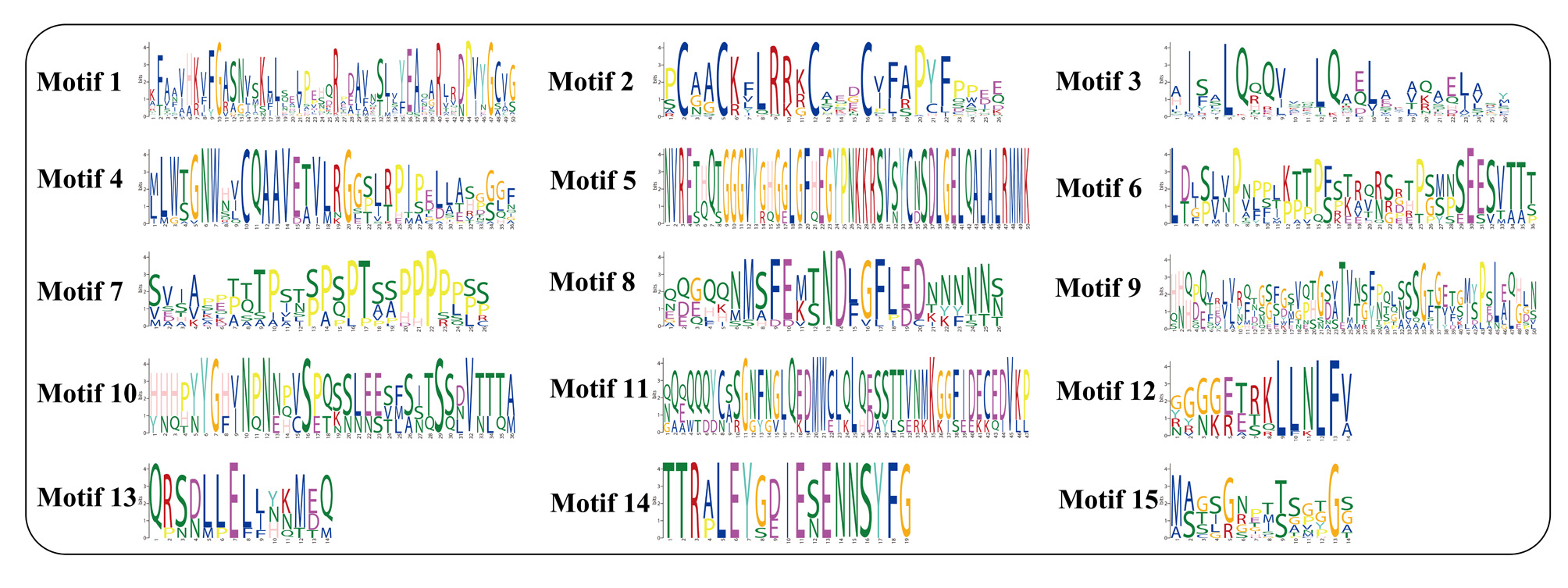

Supplement: Supplementary file 1 [file genes-11-00280-s001.zip › Additional files-20200306/Figure S2.jpg]

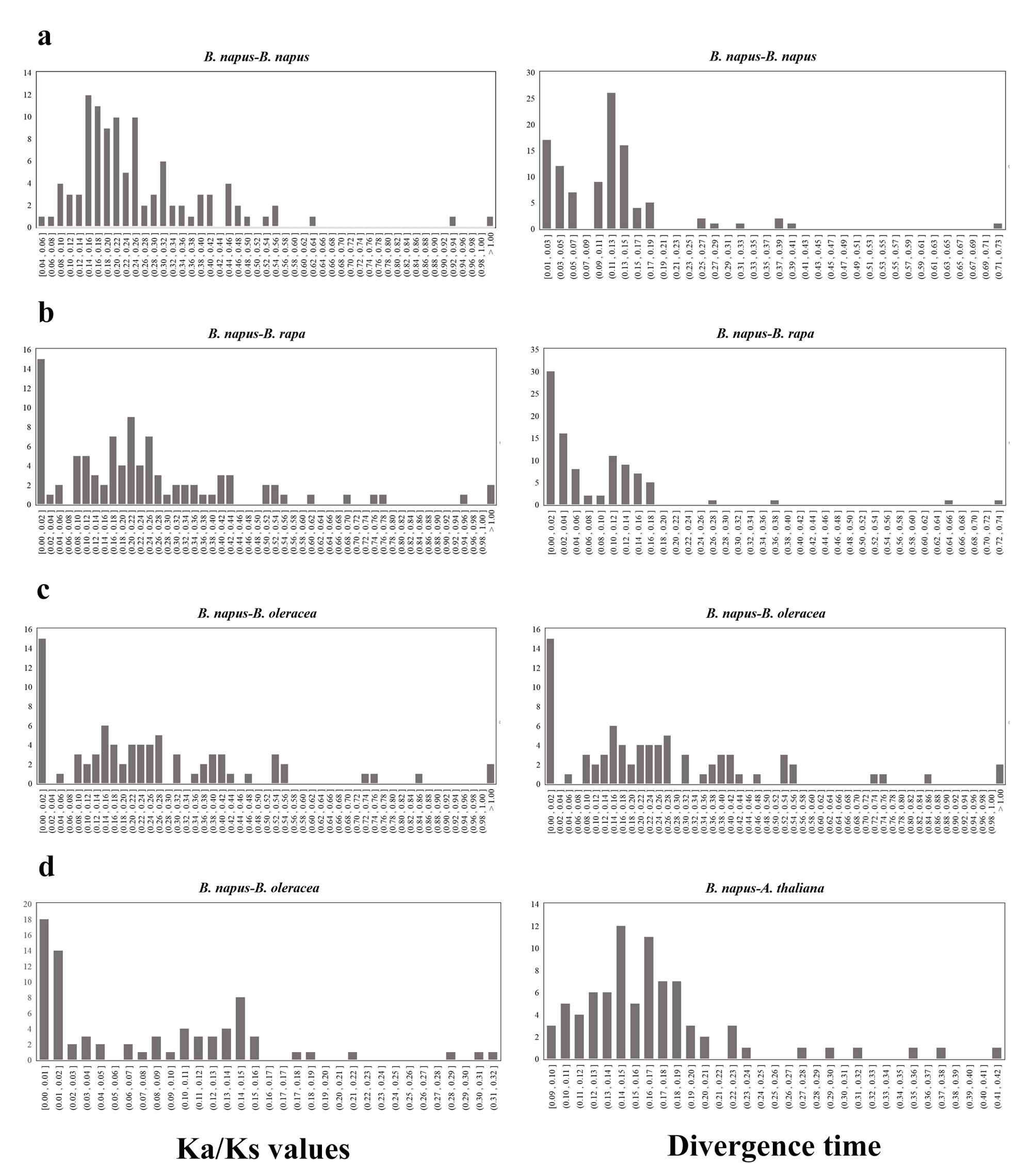

Supplement: Supplementary file 1 [file genes-11-00280-s001.zip › Additional files-20200306/Figure S3.jpg]
